# Mast Cell Degranulation Decreases Lipopolysaccharide-Induced Aortic Gene Expression and Systemic Levels of Interleukin-6 *In Vivo*

**DOI:** 10.1155/2019/3856360

**Published:** 2019-10-24

**Authors:** Jason M. Springer, Vineesh V. Raveendran, Mingcai Zhang, Ryan Funk, Donald D. Smith, Mehrdad Maz, Kottarappat N. Dileepan

**Affiliations:** ^1^Division of Allergy, Clinical Immunology and Rheumatology, Department of Medicine, University of Kansas Medical Center, Kansas City, KS, USA; ^2^Department of Cell Biology, King Faisal Specialist Hospital and Research Center, Riyadh, Saudi Arabia; ^3^Department of Orthopedic Surgery, University of Kansas Medical Center, Kansas City, KS, USA; ^4^Department of Pharmacy, University of Kansas Medical Center, Kansas City, KS, USA

## Abstract

Mast cells play an important role in immunomodulation and in the maintenance of vascular integrity. Interleukin-6 (IL-6) is one of the key biomarkers and therapeutic target in systemic vasculitis. The objective of the current study is to describe the role of mast cells in arterial IL-6 homeostasis. Eight- to ten-week-old male C57BL/6 (wild-type) mice were injected with either (a) saline, (b) compound 48/80 (a systemic mast cell degranulating agent), (c) lipopolysaccharide (LPS), or (d) a combination of C48/80 and LPS. Twenty-four hours after the injections, mice were sacrificed and serum samples and aortic tissues were analyzed for determining inflammatory response and cytokine expression profile. The results revealed that induction of mast cell degranulation significantly lowers serum IL-6 levels and aortic expression of IL-6 in LPS-treated mice. Significantly higher aortic expression of toll-like receptor-2 (TLR-2) and TNF-*α* was seen in the LPS and LPS+C48/80 groups of mice compared to controls. Aortic expression of TLR-4 was significantly decreased in LPS+C48/80 compared to C48/80 alone. LPS+C48/80-treated mice presented with a 3-fold higher aortic expression of suppressor of cytokine signaling (SOCS-1) compared to saline-injected groups. The inhibition of LPS-induced increase in serum IL-6 levels by mast cell degranulation was not seen in H1R knockout mice which suggests that mast cell-derived histamine acting through H1R may participate in the regulatory process. To examine whether the mast cell-mediated downregulation of LPS-induced IL-6 production is transient or cumulative in nature, wild-type mice were injected serially over a period of 10 days (5 injections) and serum cytokine levels were quantified. We found no significant differences in serum IL-6 levels between any of the groups. While mice injected with C48/80 or LPS had higher IL-10 compared to vehicle-injected mice, there was no difference between C48/80- and LPS+C48/80-injected mice. In conclusion, in an *in vivo* setting, mast cells appear to partially and transiently regulate systemic IL-6 homeostasis. This effect may be regulated through increased systemic IL-10 and/or aortic overexpression of SOCS-1.

## 1. Introduction

Mast cells are important cells of the immune system which exert both pro- and anti-inflammatory functions in many cell types including endothelial cells, macrophages, eosinophils, lymphocytes, and fibroblasts [1]. Because of their pluripotent modulatory functions, mast cells have been known to directly regulate a wide range of physiological functions including vasodilation, bronchoconstriction, angiogenesis [2], gastric acid production [3], atherosclerosis [4], and innate immunity [5].

Being located near the arterial walls and connective tissues, mast cells play an important, albeit complex, role in vascular homeostasis. In a mouse model of myeloperoxidase-antineutrophil cytoplasmic antibody- (MPO-ANCA-) induced vasculitis, mast cell deficiency was shown to reduce delayed hypersensitivity response to anti-MPO, regulatory T cells (Tregs), and lymph node-derived interleukin-10 (IL-10) as well as increase anti-MPO CD4^+^ T cells and enhance the disease severity [6]. Thus, mast cells appear to play a protective role in a mouse model of ANCA-associated vasculitis. However, in giant cell arteritis (GCA), a different form of systemic vasculitis, temporal artery biopsies collected from patients have shown increased number of mast cells in all layers of the vessel wall closely associated with CD3^+^ T cells and neointimal neovessels [7], suggestive of a pathogenic role of mast cells. These apparent contradictory findings of mast cells in systemic vasculitis are not well understood. However, recognizing the fact that mast cells exert both pro- and anti-inflammatory effects [1, 8], it is possible that the increased number of mast cells in the vessels during the pathogenesis of vasculitis may be a protective mechanism.

A considerable amount of literature indicates the importance of interleukin-6 (IL-6) in the pathogenesis of large vessel vasculitis. In GCA, systemic levels of IL-6 have been shown to be a predictable biomarker of active disease compared to traditional biomarkers such as the erythrocyte sedimentation rate and C-reactive protein [9]. Furthermore, inhibition of IL-6 has been shown to be an effective therapeutic strategy for the management of large vessel vasculitis [10, 11].

The objective of this study was to determine the potential role of mast cells in the regulation of vascular expression of IL-6 and the resultant changes in the levels of systemic IL-6 in a mouse model challenged with LPS. The results demonstrate that mast cell degranulation can reduce LPS-induced overexpression of IL-6 in the vascular tissue with a consequent decrease in circulating levels of IL-6. Thus, although these findings are preliminary, targeting mast cells may be considered as another therapeutic strategy for the management of vascular inflammation.

## 2. Methods

### 2.1. Chemicals

Lipopolysaccharide (LPS) and compound 48/80 (C48/80) were obtained from Sigma-Aldrich (St. Louis, MO). IL-6 and IL-10 ELISA kits were purchased from R&D Systems (Minneapolis, MN). The LPS and C48/80 stock solutions were prepared in pharmaceutical-grade sterile normal saline and subsequently diluted in sterile phosphate-buffered saline (PBS) as required.

### 2.2. Animals and Treatments

All animal experiments described in this report were approved by the Institutional Animal Care and Use Committee at the University of Kansas Medical Center in compliance with federal and state laws and regulations. The experiments utilized 8- to 10-week-old wild-type (WT) male C57BL/6 mice, (stock number 000664, Jackson Laboratory, Bar Harbor, ME). Histamine receptor KO (H1R^−/−^) breeding pairs were graciously provided by Professor Cory Teuscher of the University of Vermont. H1R^−/−^ mice colonies were subsequently maintained in our animal facility.

### 2.3. Study Design

**Figure 1:**
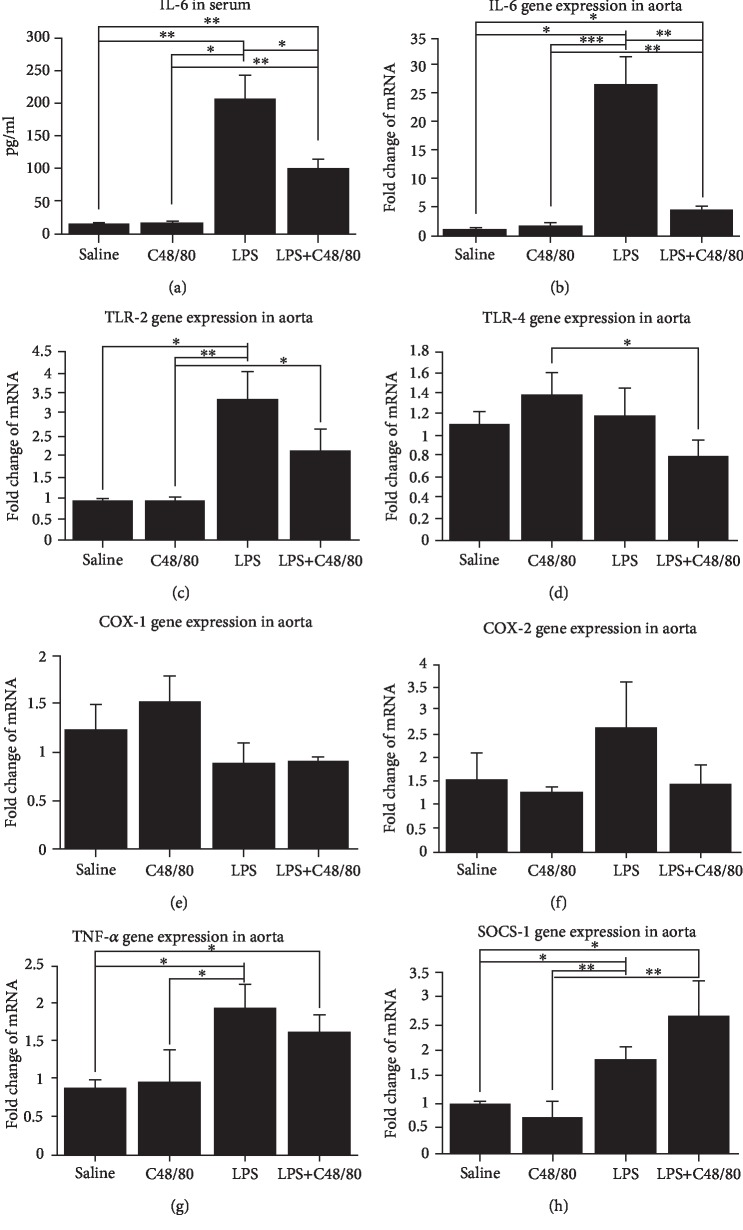
Mast cell degranulation in LPS-injected wild-type mice at 24 hours: serum levels IL-6 and aortic expressions of IL-6, TLR-2, TLR-4, COX-1, COX-2, TNF-*α*, and SOCS-1 mRNA at 24 hours. Wild-type mice 24 hours after injections with either saline, compound 48/80 (systemic mast cell degranulating agent), LPS, or LPS+C48/80. (a) LPS-induced serum IL-6 was inhibited by the administration of C48/80 (*n* = 4 per group). Aortic mRNA expression of IL-6 (b), TLR-2 (c), TLR-4 (d), COX-1 (e), COX-2 (f), TNF-*α* (g), and SOCS-1 (h) determined by qPCR. The expression levels in the saline group were normalized to 1. LPS-induced aortic expression of IL-6 was inhibited by the administration of C48/80. SOCS-1 expression, an intracellular inhibitor of IL-6 signaling, was highest in the LPS+C48/80 group (*n* = 2 in the saline group, *n* = 4 in the C48/80 group, *n* = 3 in the LPS group, and *n* = 3 in the LPS+C48/80 group). ^∗^*p* < 0.05 and ^∗∗^*p* < 0.01.

**Figure 2:**
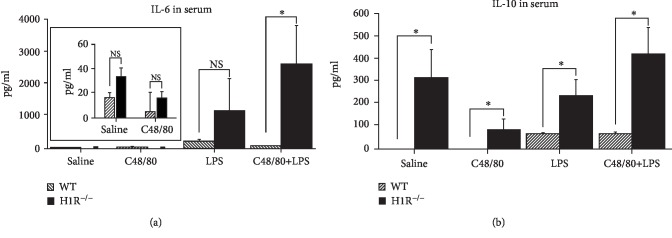
Mast cell degranulation in H1R^−/−^ mice at 24 hours: wild-type mice (shaded columns; *n* = 12 mice per group) and H1R^−/−^ mice (black columns; *n* = 8 mice per group) were treated with (i) saline, (ii) compound 48/80 (a mast cell degranulating agent), (iii) LPS, or (iv) LPS and C48/80. (a) Unlike in the wild-type mice, there were no significant differences between any of the groups in H1R^−/−^ mice at 24 hours. Inset depicts enlarged presentation of data for saline and C48/80 treatment. Note the scale on *y*-axis. (b) H1R^−/−^ mice demonstrated higher serum IL-10 in the C48/80 and LPS groups compared to saline-injected mice at 24 hours. ^∗^*p* < 0.05 and ^∗∗^*p* < 0.01.

**Figure 3:**
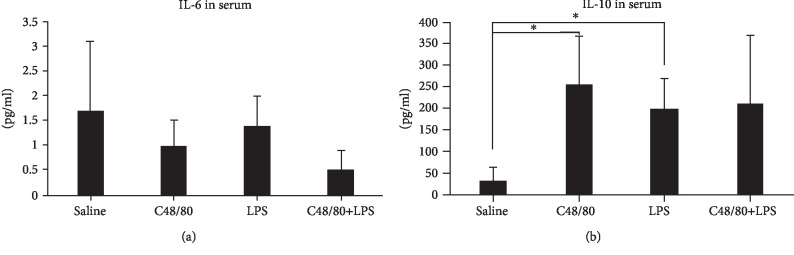
Subacute effects of mast cell degranulation (10 days): wild-type mice injected serially over a period of 10 days had low levels of serum IL-6 without any difference among the groups (a). Serum IL-10 levels remained elevated at 10 days in the C48/80, LPS, and C48/80+LPS groups (b). (*n* = 4 per group.) ^∗^*p* < 0.05 and ^∗∗^*p* < 0.01.

Both genotypes of mice were separately assigned into four groups by random selection as follows: “saline”; “compound 48/80 (C48/80),” a pharmacological agent that induces systemic mast cell degranulation; “LPS”; and “C48/80+LPS.” The selected concentrations of C48/80 and LPS were based on prior experience of animal tolerance and reproducibility of response [4, 12]. Mice were intraperitoneally injected with phosphate-buffered saline (PBS) (vehicle solution), C48/80 (1 mg/kg, body weight (b.w.)), LPS (1 mg/kg b.w.), or a combination of C48/80 (1 mg/kg b.w.)+LPS (1 mg/kg b.w.). In the first set of experiments (Figure 1), wild-type mice were sacrificed 24 hours after 1 injection (*n* = 4 per group). In the second set of experiments (Figure 2), in addition to wild-type mice, H1R^−/−^ mice received the same treatments and were sacrificed 24 hours later. In the final set of experiments (Figure 3), wild-type mice were injected every other day with the same treatments for a period of 10 days (5 total injections). All animals had free access to drinking water and food *ad libitum*.

### 2.4. Collection of the Blood and Aorta

Blood samples were collected from the retroorbital sinus under anesthesia at the time of necropsy. Mice were euthanized using isoflurane (Abbott, North Chicago, IL) inhalation followed by opening the thoracic cavity. The aorta was collected and stored in RNA later until used for the gene expression studies.

### 2.5. Messenger RNA Expressions in the Aorta

Total RNA was extracted from the aorta using TRIzol Reagent. Total RNA was reverse transcribed into first-strand cDNA using a High-Capacity cDNA Reverse Transcription Kit (Applied Biosystems, CA) following the manufacturer's recommendations. Quantitative real-time RT-PCR was performed using the ABI7500 Real-Time PCR system (Applied Biosystems). The amplification reactions were performed in 25 *μ*l total volume containing SYBR Green PCR Master Mix with respective primers and 5 *μ*l of cDNA of each mouse. The primers were designed using Primer Express Software v3.0 (Applied Biosystems). The mRNA expressions of genes of each sample were normalized with their mRNA expression of *β*-actin. After normalization, the fold change of mRNA of each gene relative to one sample from the control group was calculated by comparative *ΔΔ*CT method. For the first set of experiments, aortic tissue was available for 2 in the saline group, 4 in the C48/80 group, 3 in the LPS group, and 3 in the LPS+C48/80 groups.

### 2.6. Serum IL-6 and IL-10 Measurement

Serum was isolated from blood samples and stored at -80°C until their use. The cytokine levels were quantified by using ELISA kits (R&D Systems) according to the manufacturer's recommendations.

### 2.7. Statistical Analysis

Student's *t*-test, one-way or two-way ANOVA was adopted for statistical analyses, as applicable. Results are presented as mean ± SEM and *p* < 0.05 is considered as significant. GraphPad Software v5.04 (San Diego, CA) was used to perform all the statistical analyses.

## 3. Results

### 3.1. Mast Cell Degranulation Inhibits LPS-Induced Aortic Gene Expression and Circulating Levels of IL-6 at 24 Hours

At 24 hours, induction of mast cell degranulation with C48/80 alone did not affect the basal level of either serum IL-6 or IL-6 mRNA expression in aortic tissue compared to controls injected with PBS. In contrast, LPS injections resulted in a 13-fold increase in serum IL-6 as well as a 26-fold increase in aortic IL-6 gene expression. Interestingly, mast cell degranulation reduced LPS-induced serum IL-6 levels by approximately 50% and caused approximately an 80% decrease in aortic IL-6 expression (Figures 1(a) and 1(b)). In contrast, the aortic mRNA expressions of TNF-*α* were higher in both LPS- and LPS+C48/80-treated mice (Figure 1(g)).

Toll-like receptor-2 (TLR-2) and TLR-4 have been shown to be ubiquitously expressed throughout the human aorta [13] as well as upregulated by histamine on human vascular endothelial cells [14]. TLR-4 stimulation by LPS has been shown to induce a transmural panarteritis in human temporal arteries engrafted into SCID mice [15]. Therefore, the effects of the aortic expression of these TLRs by mast cell degranulation are of interest. At 24 hours, aortic expression of TLR-2 in LPS-treated mice was 3-fold higher when compared to those injected with PBS or C48/80. There were no significant differences in aortic TLR-2 expression between mice injected with LPS and LPS+C48/80-injected mice (Figure 1(c)). The levels of expression of TLR-4 in the aorta were lower in LPS+C48/80 compared to C48/80 alone, but otherwise, there were no differences among the groups (Figure 1(d)). The underlying mechanism(s) for this counterintuitive effect of LPS on the expression of TLR-2 or TLR-4 in aorta tissue is currently unknown. It is possible that this may be from the activation of different downstream pathways upon LPS stimulation [16].

Cyclooxygenase-2 (COX-2) is an important marker of inflammation and is induced by bacterial agents, cytokines, and vasodilatory molecules. Our previous work has demonstrated that histamine, a major mast cell-derived vasoactive mediator, could induce COX-2 expression in human coronary artery endothelial cells *in vitro*, directly and synergistically with LPS [17]. However, in these *in vivo* experiments, we did not notice any significant differences in the expression of either COX-1 or COX-2 mRNA within these four treatment groups at 24 hours (Figure 1(e)–1(f)), indicating the involvement of the cells in the arterial wall other than endothelial cells, such as residential macrophages and/or smooth muscle cells, in the regulation of COX-2 expression in aortic tissue upon mast cell activation [1].

SOCS-1 has been shown to selectively inhibit LPS-induced IL-6 signaling through JAK-STAT pathways [18]. We therefore hypothesized that inhibition of LPS-induced IL-6 production could be mediated through increased expression of SOCS-1. As shown in Figure 1(h), aortic expression of SOCS-1 mRNA was significantly higher in LPS-treated mice compared to PBS at 24 hours. The LPS+C48/80 group had a significantly higher SOCS-1 mRNA aortic expression compared to the PBS and C48/80 groups at 24 hours. Although the SOCS-1 expression was higher in the LPS+C48/80 group compared to LPS alone, this did not reach statistical significance.

### 3.2. Mast Cell Regulation of Systemic IL-6 Involves H1R at 24 Hours

It is well-established that histamine is a major constituent of the mast cell and is instantaneously released during mast cell degranulation and that histamine induces endothelial cell activation in the vasculature via histamine H1 receptors (H1R) [19]. To examine whether mast cell degranulation-mediated effects presented here are mediated through H1R, we tested the effects of LPS, C48/80, and LPS+C48/80 in H1R knockout (H1R^−/−^) mice at 24 hours. In comparison to wild-type mice, mast cell degranulation did not significantly affect LPS-induced IL-6 production in H1R^−/−^ mice (Figure 2(a), black columns). Between the wild-type mice and the H1R^−/−^ mice, there was no difference in serum IL-6 in mice injected with PBS or C48/80. With LPS alone, there was over a 5-fold difference between the wild-type mice and H1R^−/−^ mice although this did not reach significance due to the large standard deviation in the H1R^−/−^ group. Serum IL-6 level was increased by 35-fold in H1R^−/−^ mice injected with C48/80 along with LPS compared to their respective wild-type counterpart (*p* = 0.016). Systemic IL-6 was not significantly different among all the H1R^−/−^ groups (Figure 2(a)). In all groups, there was significantly higher IL-10 in H1R^−/−^ compared to controls. There was significantly higher IL-10 in H1R^−/−^ mice receiving LPS+C48/80 compared to C48/80 alone (*p* = 0.027) (Figure 2(b)). Thus, the modulatory effects of mast cell degranulation on LPS-induced serum IL-6 are abrogated in the absence of the H1R receptor suggesting the importance of histamine signaling in the regulatory process. It is noteworthy that despite the variant cellular sources of IL-10 [20], a potent anti-inflammatory cytokine, IL-10 is also present in mast cells [21].

### 3.3. Mast Cell-Mediated Inhibition of LPS-Induced Systemic IL-6 Is Lost and Is Associated with Elevated Levels of Systemic IL-10 at 10 Days

To test the long-term effects of mast cell degranulation on LPS-induced inflammatory responses, we monitored the levels of IL-6 and IL-10 in wild-type mice that received 5 alternate-day injections over a 10-day period (Figures 3(a) and 3(b)). After the 10-day treatment, there were no significant differences in serum IL-6 levels between groups of mice. When compared to PBS-treated controls, mice injected with C48/80 or LPS had their serum IL-10 levels increased by 3.5-fold and 6-fold, respectively. Furthermore, no significant differences in IL-10 levels were noted between the LPS and LPS+C48/80 groups. Thus, the primary regulatory effects of mast cells on aortic IL-6 production appear to be an acute effect, supporting the role of mast cells in the innate immune response.

## 4. Discussion

LPS, an endotoxin produced by Gram-negative bacteria, acts through TLR-4 complexes to produce systemic inflammation. LPS is a known inducer of aortic IL-6 production [21]. Our results demonstrate that mast cell degranulation could effectively inhibit LPS-induced aortic IL-6 gene expression and its circulating levels (Figures 1(a) and 1(b)). Prior work done by our group has demonstrated that *in vitro* mast cell degranulation synergistically enhances LPS-induced IL-6 production in human aortic endothelial cells [22]. Taken together, this suggests that mast cells may regulate other cells within the artery such as macrophages or smooth muscle cells [1].

The inhibition of LPS-induced IL-6 production by mast cells appears to be an acute effect. These effects were evident 24 hours after the injections (Figure 1(a)). Prior work by our group has demonstrated that the LPS-induced systemic levels of IL-6 peak around 3-6 hours with a progressive decline afterwards (unpublished data). Repeated daily injections of LPS to wild-type mice did not elevate the IL-6 over a 10-day time course (Figure 3(a)). Furthermore, the mast cell-mediated downregulation of LPS-induced IL-6 synthesis was not seen in mice that received daily injections of LPS for 10 days. It is known that continuous exposure of LPS leads to desensitization. The present findings are in agreement with this contention and suggest that the lack of LPS responsiveness in mice continuously exposed to LPS for 10 days is due to desensitization-associated tolerance, potentially due to mast cell-mediated modulation of innate immune responses.

During degranulation, mast cells exocytose granules which contain a variety of preformed and newly synthesized immunomodulatory agents. These include, but are not limited to, histamine, proteases, many cytokines, and proteoglycans as well as cyclooxygenase and lipoxygenase products [1]. We have extensively studied the influence of the mast cell-derived histamine-H1R-IL-6 axis by human endothelial cells [14, 17, 23, 24]. Therefore, it was of interest to examine the potential involvement of H1R in our in vivo system. As shown in Figure 2(a), the inhibitory effects of mast cells on IL-6 was lost in H1R^−/−^ mice suggesting that histamine-H1R signaling may be an integral component in the regulation of IL-6 homeostasis *in vivo*. Nevertheless, it should be emphasized that there are four major histamine receptors [25, 26] and we have not attempted to identify the potential participation of H2, H3, and H4 receptors in this study. Another group has made the observation that deletion of H1R in nasal fibroblasts results in inhibition of histamine-induced IL-6 [27]. This lends support to the fact that H1R regulates IL-6, but the effects may be cell specific.

IL-10 is a well-recognized anti-inflammatory cytokine [28–30], and mast cells release IL-10 during degranulation. In the current study, increased IL-10 levels, which may not solely be released from mast cells [20], may also play an immunoregulatory role in inhibiting IL-6 production. It is of interest that mast cell degranulation, in combination with LPS, increased aortic expression of SOCS-1, an intracellular protein acting in a negative feedback loop in IL-6 signaling, which was associated with reduction in LPS-induced IL-6 generation in mice treated with C48/80. This increase in SOCS-1 may play a role in the mast cell-mediated reduction in the aortic expression of IL-6 and systemic levels of IL-6 in mice treated with LPS and C48/80. Further studies are warranted to elucidate the existence of the histamine-H1R-SOCS-1 axis in IL-6 homeostasis in autoimmune diseases and sepsis. Given that the peripheral blood mononuclear cells (PBMCs) also produce IL-6 and IL-10, we could not rule out the contribution of PMBCs to the observed changes in serum IL-6 and IL-10 in the current study.

In conclusion, mast cells appear to play an important role in the regulation of the IL-6 gene expression and cytokine production *in vivo*. We speculate that this regulatory effect may be attributed to the increased expression of SOCS-1 even though the link between the H1R signaling pathway and SOCS-1 has not been clearly established. This adds to the growing body of literature supporting the role of mast cells as an immunomodulator in systemic forms of vasculitis.

## Data Availability

The data used to support the findings of this study are included within the article. Additional questions can be addressed to the corresponding author.
